# Ultrasound-assisted synthesis of N235-impregnated resins for vanadium (V) adsorption

**DOI:** 10.1098/rsos.171746

**Published:** 2018-04-25

**Authors:** Bo Chen, Shenxu Bao, Yimin Zhang, Ruwei Zheng

**Affiliations:** 1 School of Resources and Environmental Engineering, Wuhan University of Technology, Wuhan 430070, People's Republic of China; 2 Hubei Key laboratory of Mineral Resources Processing and Environment, Wuhan 430070, People's Republic of China; 3 Hubei Collaborative Innovation Center for High Efficient Utilization of Vanadium Resources, Hubei Provincial Engineering Technology Research Center of High Efficient Cleaning Utilization for Shale Vanadium Resource, Wuhan University of Science and Technology, Wuhan 430081, People's Republic of China

**Keywords:** solvent-impregnated resins, ultrasound impregnation, vanadium, adsorption, stability

## Abstract

N235-impregnated resins were prepared using XAD-16HP macroporous adsorption resins as support with and without ultrasonic irradiation to evaluate the effects of ultrasound impregnation (UI) on the preparation and adsorption characteristics of the resins. The results show that the impregnation ratio of the solvent-impregnated resins (SIRs) prepared by ultrasound impregnation method (SIRs-UI) increases obviously but their adsorption capacity for V(V) just slightly increases and the utilization rate of the extractant decreases with the augmentation of ultrasound power. This may be caused by the fact that more extractant can enter into the deeper pores of the resins under high ultrasound intensity, but these extractants cannot effectively react with V(V). The impregnation equilibrium time of SIRs-UI can be obviously shortened in comparison to that of the SIRs prepared by conventional impregnation method (SIRs-CI) (3 min versus 240 min) due to the cavitation effect evoked by ultrasound. Ultrasonic irradiation may cause more N235 desorbed from the pores of the resin at low N235 content, resulting in lower adsorption capacity for V(V) than that of SIRs-CI, but the adsorption capacity is inverse at higher N235 content. N235 may be distributed more homogeneously in the pores of XAD-16HP with ultrasonic irradiation, thus, SIRs-UI presents higher adsorption capacity and stronger stability than SIRs-CI. This study manifests that ultrasound-assistant impregnation method may be a potential and promising technique for the preparation of SIRs.

## 1. Introduction

Vanadium is an important strategic metal element, which is extensively applied in numerous fields due to its unique properties, such as high melting point, fatigue resistance and tensile strength [1–4]. Solvent-impregnated resins (SIRs) have been applied to separate and purify vanadium from vanadium-containing solutions by researchers on account of their distinctive selectivity, easy operability and environmental friendliness [5,6]. The work of separating and purifying metal ions and organics from solutions by SIRs can be traced back to the principal and pioneering work conducted by Warshawsky [7], Grinstead [8] and Kroebel & Meyer [9]. As is known to us, SIRs are generally prepared by incorporating extractant into macroporous polymer support via a physical impregnation technique [10,11]. The conventional impregnation (CI) methods for the preparation of SIRs in the laboratory are usually conducted by a constant temperature bath oscillator [5,6,12] or magnetic stirrer [13]; however, these CI processes suffer from being time- and energy-consuming, which may impede the industrial application of SIRs. For instance, the impregnation time for the SIRs containing Cyanex 923 prepared by Nguyen *et al*. [14] is about 24 h and that for the D2EHPA impregnated resins prepared by Liang *et al*. [5] also needs about 24 h (table 1).

**Table 1.** Nomenclature.

symbols	
*η*	impregnation ratio of the SIRs, %
*m*_2_	weight of the dry SIRs, g
*m*_1_	weight of the treated dry resins, g
*Q*_M_	adsorption capacity of SIRs for V(V), mg g^−1^
*C*_e_	equilibrium concentration of V(V), mg l^−1^
*C*_0_	initial concentration of V(V), mg l^−1^
*Q*_e_	equilibrium adsorption capacity of SIRs for V(V), mg g^−1^
*Q*_m_	saturated adsorption capacity of SIRs for V(V), mg g^−1^
*K*_L_	empirical parameter of Langmuir isotherm model
*K*_F_	constant of Freundlich isotherm model
*Q*_M_/*η*	the utilization rate of loaded N235

In recent years, new applications and effects of ultrasound on chemical, physical, mechanical changes of materials have drawn great attention, particularly in the preparation of catalysts and absorbents. Ding *et al*. [15] fabricated carbon nitride nanosheets decorated with WO_3_ nanorods (WO_3_/g-C_3_N_4_) via ultrasonic-assisted dispersion and conventional incipient wetness impregnation method. They found that ultrasound promoted the formation of g-C_3_N_4_ nanosheets efficiently and the novel WO_3_/g-C_3_N_4_ nanocomposites show excellent catalytic performance and super stability. Kumar *et al*. [16] applied ultrasound irradiation to ZSM-5 and beta zeolite for the synthesis of Pt-modified catalysts. The results revealed that the catalysts prepared by *in situ* synthesis with ultrasound irradiation have higher conversion of *n*-pentane and selectivity to iso-pentane than those prepared without ultrasound irradiation. Xiao *et al*. [17] synthesized Ag/Cu/Fe-supported activated carbon (AC) by ultrasonic-assisted impregnation method, and concluded that the application of ultrasound irradiation to prepare metal ion-impregnated AC can make the metallic particles on the carbon surfaces become finer and disperse better in comparison with the use of impregnation only, which leads to the result that the Ag/AC and Cu/AC prepared by ultrasound irradiation have higher adsorption capacities for DBT than the Ag/AC and Cu/AC prepared using impregnation only. In sum, the application of ultrasound impregnation (UI) method can bring lots of beneficial effects, such as improving the performance and stability of the catalysts, improving the conversion and selectivity of the catalysts, making the surface of AC fine, enhancing the adsorption capacity of the ion-impregnated AC for DBT. Effects evoked via ultrasound in aqueous solutions are ascribed to acoustic cavitation including the generation, growth and collapse of bubbles in solutions [18]. Extreme high temperature and pressure are produced during the collapse of cavitation bubbles [19], which not only bring about a mechanical action on liquid–solid interface, but also chemical and physical effects [20,21].

To our best knowledge, there are few published papers in open literature on the use of ultrasonic irradiation in the preparation of SIRs and the adsorption characteristics for metals of such prepared SIRs. In our previous work, N235-impregnated resins were prepared by CI method and applied to separate and purify V(V) from vanadium-containing solutions due to their high affinity to V(V) in low pH environment [22]. In this study, an UI technique is put forward to prepare N235-impregnated resins. The objective of this work is to introduce a novel method into the preparation process of SIRs and probe the performance of such prepared SIRs by comparing the impregnation process and adsorption characteristics for V(V) of the SIRs prepared by CI and UI methods.

## 2. Experimental set-up

### 2.1. Materials

Macroporous resin Amberlite® XAD-16HP, supplied by Shanghai Anland Co., Ltd, China, was used as the support for the preparation of SIRs. The physical characteristics of XAD-16HP are listed in table 2. First, the resin was soaked in ethanol for 4 h to remove the remained monomers and other types of impurities produced in the fabrication process, followed by washing with deionized water, and then dried at 60°C in a vacuum oven for 12 h before the preparation of SIRs.

**Table 2.** Physical characteristics of XAD-16HP resin.

parameters	value
specific surface area	800 m^2^ g^−1^
pore volume	1.82 ml g^−1^
average pore diameter	15 nm
particle size	0.425–0.850 mm

Trialkylamine (N235, R_3_N, R = C_8_–C_10_) was purchased from Qinshi Technology Co., Ltd, China. The petroleum ether with boiling point range of 60–90°C was used as diluent to dilute N235 during the SIRs preparation process. Acidic vanadium solutions were prepared by dissolving V_2_O_5_ (analytical grade, received from Shanghai Hushi Laboratorial Equipment Co., China) in H_2_SO_4_ solutions. Other chemicals used in this study were of analytical grade.

### 2.2 Preparation of solvent-impregnated resins

**Figure 1.**
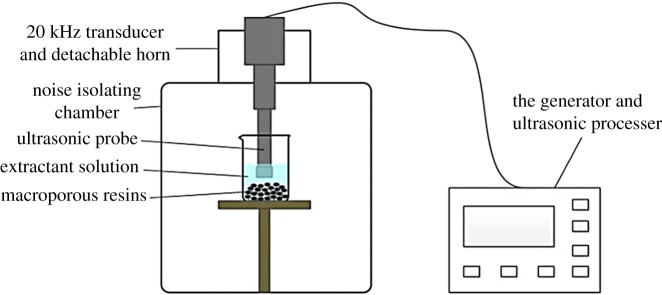
Schematic of ultrasonic impregnation instrument.

The UI experiments were conducted by using an ultrasonic dispersion apparatus, as shown in figure 1. The 20 kHz ultrasonic irradiation was carried out with a commercial supply ultrasonic generator (CP505, Vernon Hills, Illinois, USA) equipped with a solid ultrasonic probe installed at the top of the noise isolating chamber.

N235 was firstly diluted in petroleum ether, and then the diluted N235 was mixed with the pretreated XAD-16HP resins (as the liquid–solid ratio of 30 : 1 ml g^−1^). Subsequently, the ultrasonic probe was inserted into the N235 solutions to irritate the mixture with different ultrasonic power, impregnation time and extractant concentration. After impregnation, the resins were separated by filtration using a Buchner funnel and then washed with deionized water until the eluent was clear. Finally, the N235-impregnated resins were heated at 60°C in a vacuum oven for 12 h to remove the remained diluent prior to use. The impregnation ratio of the SIRs, η(%), i.e. the ratio of the loaded extractant to the support, was calculated as follows:

2.1 $$\eta =\frac{m_{2}-m_{1}}{m_{2}}\times 100\%,$$

where *m*_2_ is the weight of the dry SIRs (g) and *m*_1_ is the weight of the pretreated dry resins (g).

The CI process for N235-impregnated resins is similar to that in the UI experiment. The difference is that the impregnation process of the latter was conducted in a constant temperature bath oscillator (SHA-2, Jintan Yitong Electronic Co., Ltd, China) at the following conditions: stirring rate of 120 r.p.m., temperature of 25°C and liquid–solid ratio of 30 : 1 ml g^−1^.

### 2.3 Static adsorption experiments

The pH of the aqueous solutions containing different concentrations of V(V) was adjusted to 1.8 prior to use. First, the dry SIRs were added to V(V) solutions (as the liquid–solid ratio of 40 : 1 ml g^−1^) in a conical flask, then the mixture was shaken (120 r.p.m.) at a bath oscillator at 25°C for different contacting time. Subsequently, the mixture was filtrated and the adsorption capacity of SIRs for V(V), *Q*_M_ (mg g^−1^), was calculated according to

2.2 $$\;Q_{M}=\frac{(C_{0}-C_{e})V}{m_{2}},$$

where *V* is the volume of V(V) solutions (litres), *C*_0_ and *C*_e_ are initial and equilibrium concentration of V(V) in the solutions (mg l^−1^), respectively and *m*_2_ is the weight of the dry SIRs (g).

### 2.4 Cyclic adsorption–desorption experiments

The N235-impregnated resins were separated from the V(V) solutions after adsorption equilibrium, and then they were added to 20 ml Na_2_CO_3_ solutions with a mass concentration of 14%. After shaking for 8 h at room temperature, the SIRs were filtered and washed with dilute sulfuric acid (pH = 1.8) until the pH value of the eluent was about 1.8 and were re-used for the next static adsorption for V(V), the process of which was the same as that depicted in section ‘Static adsorption experiments’.

### 2.5 Measurements

The concentration of vanadium in the solution was determined by ferrous ammonium sulfate titration using 2-(phenylamino)-benzoic acid as indicator [23]. In order to observe the vanadium distribution in the SIRs, SIRs-UI and SIRs-CI after adsorption of vanadium were carefully split by blade from the core and the cross section of resins were inspected and scanned by scanning electron microscopy (SEM, JSM-IT300, JEOL, Japan) equipped with an energy dispersive spectrometer (EDS, Oxford, UK).

## 3. Results and discussion

### 3.1. Effects of ultrasonic conditions on solvent-impregnated resins

#### 3.1.1. Ultrasonic power

**Figure 2.**
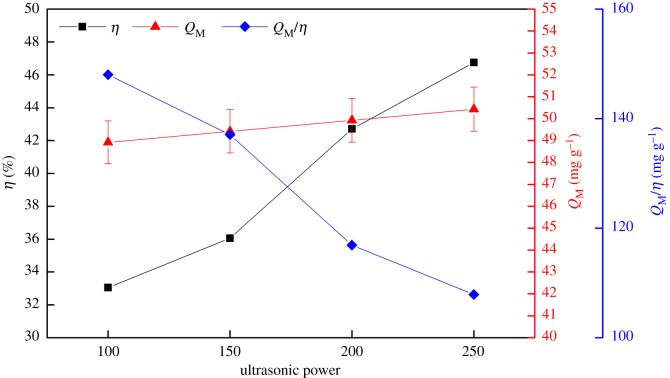
Effects of ultrasonic power on preparation of SIRs and adsorption capacity.

**Figure 3.**
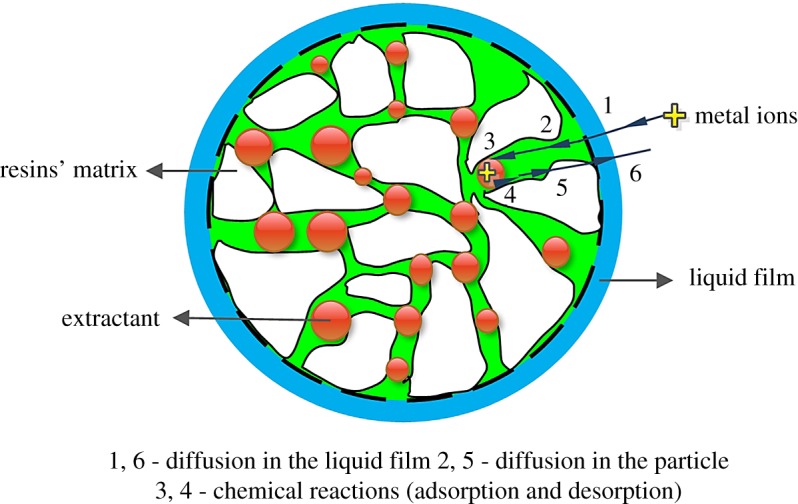
The schematic for the working of SIRs.

The effects of ultrasonic power on the preparation of SIRs and adsorption capacity for V(V) under the conditions of 50% N235 and 5 min impregnation time are shown in figure 2. The schematic to explain the working of SIRs is shown in figure 3.

It is seen from figure 2 that the impregnation ratio of the SIRs prepared by ultrasound impregnation method (SIRs-UI) obviously increases with the augmentation of ultrasonic power, indicating that ultrasonic irradiation can enhance the impregnation of extractant on macroporous resins. The action modes of ultrasound on impregnation of extractant are related to cavitation phenomenon, in which bubbles are nucleated and collapsed in the liquid phase during ultrasonic irradiation. The asymmetric or symmetric collapse can lead to high-speed microjets and microscopic turbulent flow or microstreaming at the liquid–solid interface between the extractant and macroporous resins. Cavitation collapse enhances the contact and spread of extractant on the surface of pores in support resins [24], usually enhanced with the increase of the temperature, by generating local high temperature and pressure near it. It is clear that the higher the ultrasound power is, the greater the intensity of ultrasonic field can be obtained. Thus, the number of cavitation events, high-speed microjets and microstreaming were enhanced, which led to more extractant entering the pores and increasing the impregnation ratio of the SIRs. With the increase of loaded N235 on the resin, more extractant can react with V(V) and thus the adsorption capacity also increases (figure 2). However, it is easy to understand from the adsorption process of metals onto SIRs (figure 3) that as more N235 goes into the deeper pores at the higher ultrasound power, the extractant in the deeper pores is hard to react with V(V) under the adsorption time of 12 h, resulting in the slight increase of the adsorption capacity and the decline of the utilization rate of N235 (*Q*_M_/*η*), which indicates that the percentage of the effective N235 in adsorbing V(V) decreases with the increase of the impregnation ratio of the SIRs (figure 2). As a result, taking both the utilization rate of N235 and the adsorption capacity for V(V) into consideration, the optimal ultrasonic power was determined as 100 W for the preparation of SIRs in the following experiments.

#### 3.1.2. Impregnation time

**Figure 4.**
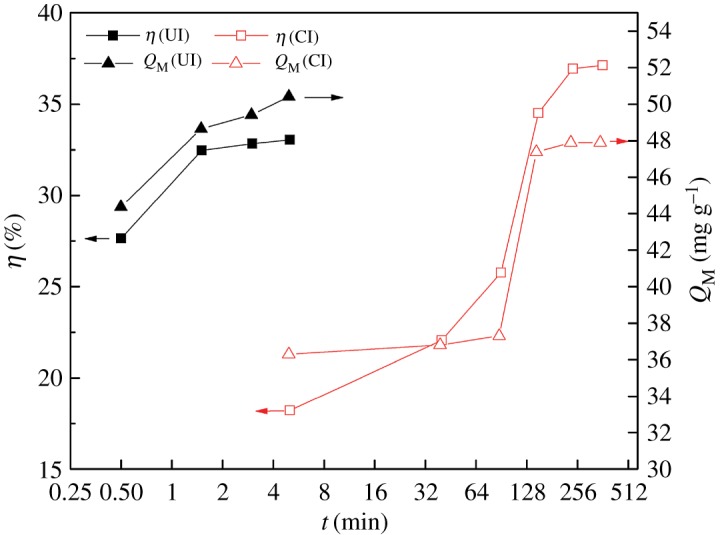
Effects of impregnation time on preparation of SIRs and adsorption capacity.

The effects of impregnation time on the preparation of the SIRs using UI and CI methods, respectively, and their adsorption capacity for V(V) are compared under the conditions of 50% N235 and 100 W ultrasonic power in figure 4.

Figure 4 shows that the impregnation ratio of both SIRs increases with the increasing impregnation time, and the impregnation equilibrium time for the SIRs-UI (approx. 3 min) is by far shorter than that for the SIRs prepared by CI method (SIRs-CI) (approx. 240 min), verifying the superiority of ultrasonic irradiation over CI on less time-consuming [25]. However, the equilibrium impregnation ratio of the former is lower than that of the latter. It indicates that ultrasound can significantly accelerate the impregnation process but may cause some parts of the extractant loaded on the resin to be desorbed and enter the aqueous phase [26]. As mentioned above, when the cavitation bubbles caused by ultrasound collapse asymmetrically or symmetrically, local high temperature and high pressure are generated and then high-speed microjets and shock waves are triggered in the fluid [20], which can largely enhance the mass transfer of the extractant on the support and then reduce the equilibrium time [27]. However, the high-speed microjets may incessantly impinge the resin surface and the shock waves produce microscopic turbulence or microstreaming at interfacial films surrounding nearby the resin particles and within the pores. Thus, this action may break some bonds between the extractant and the resin and cause some parts of loaded extractant to get into the aqueous phase, leading to the adsorption–desorption equilibrium between the extractant and the resins. Although the amount of N235 loaded on the SIRs-UI is lower than on the SIRs-CI, the adsorption capacity of the former is higher than that of the latter for V(V) (figure 4), suggesting the different impregnation or adsorption status of the extractant onto the resin. According to our previous study [6], the extractant is preferentially impregnated in the micropores as wall-spreading approximately to form monolayer films, but it is more easily accumulated in the macropores and/or mesopores as pore-filling mechanism to form multilayer during CI process. XAD-16HP resin used in this study is mainly composed of macropores and mesopores, and the extractant is likely to accumulate in the pores as multimolecular layers by using CI method. As UI method is applied, the cavitation effect of ultrasound enhances the spread of extractant in the pores and makes it distribute more homogeneously on the surface of the pores. Thus, more loaded extractant in the resin can effectively react with V(V), bringing about the increase of adsorption capacity though the total impregnated extractant is less in comparison with that in the SIRs-CI. This also can be used to explain the fact that the impregnation ratio of the SIRs-UI just slightly increases when the impregnation time is extended from 3 to 5 min but the improvement of the adsorption capacity is relatively obvious (figure 4). Therefore, the effect of ultrasound on impregnation process of SIRs can be simply summarized as the promotion of mass transfer and spread of the extractant on the pores surface of the resin. In order to obtain better adsorption capacity for V(V), the optimal impregnation time was selected as 5 min for UI.

### 3.2 Effects of extractant concentration

**Figure 5.**
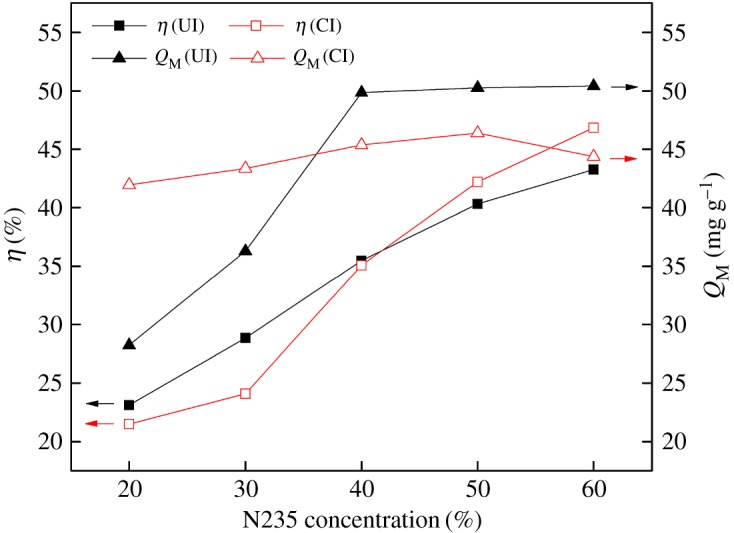
Effects of N235 concentration on preparation of SIRs and adsorption capacity.

Figure 5 shows that the impregnation ratio of SIRs-CI and SIRs-UI both monotonously increase with the increasing N235 concentration, indicating that the loaded extractant on the resins is proportional to the extractant content in the impregnation solution. As for the adsorption capacity of SIRs-CI for V(V), it slightly increases with the increasing N235 concentration. However, it is interesting to observe that the adsorption capacity of SIRs-UI for V(V) is relatively low at the lower N235 concentrations but it increases rapidly and keeps stable when the content of N235 reaches 40%. It may be speculated that this phenomenon is caused by the different adsorption–desorption equilibrium for N235 and petroleum ether under ultrasonic irradiation. Ultrasonic irradiation may enhance the desorption of N235 other than that of petroleum ether from the pores due to the viscosity discrepancy at low N235 concentrations, which results in the different N235 content in the resins, leading to distinct difference in the adsorption capacity of SIRs-UI and SIRs-CI though they have similar impregnation rate. With the increasing content of N235, more N235 can enter the pores and then adsorption capacity of SIRs-UI increases obviously (figure 5). As mentioned above, N235 is easy to accumulate in the pores of XAD-16HP to form multilayer by using CI method. Thus, the adsorption capacity of SIRs-CI just slightly increases as the effective N235 reacting with V(V) does not increases remarkably in spite of the augmentation of the loaded extractant, and it even declines a bit when the N235 content is elevated from 50 to 60%, because the pores filling rate of SIRs-CI is close to the peak value and the effective N235 loaded on SIRs-CI may somewhat decline in this condition.

### 3.3 Effects of contacting time

**Figure 6.**
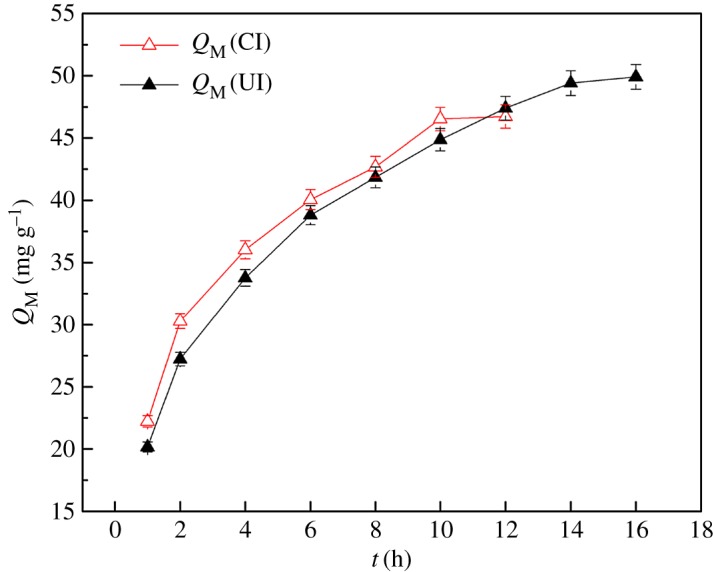
Effects of contacting time on adsorption capacity for V(V) onto SIRs.

**Figure 7.**
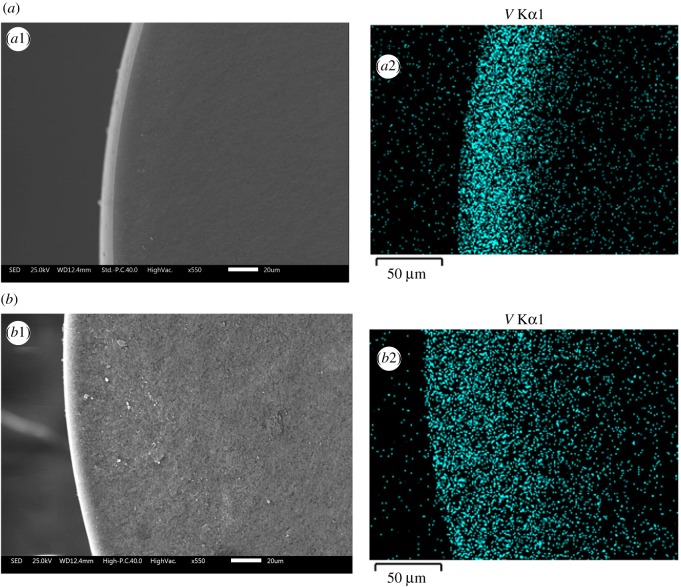
SEM-EDS plane scan analyses of the cross section of SIRs-CI (*a*) and SIRs-UI (*b*) after adsorption of V(V).

The effects of contacting time on the adsorption for V(V) with initial concentration of 1350 mg l^−1^ onto SIRs-UI and SIRs-CI, respectively, are plotted in figure 6. The SEM-EDS plane scan analyses for the cross section of SIRs-CI (*a*) and SIRs-UI (*b*) after adsorption of V(V) are shown in figure 7.

Figure 6 manifests that the adsorption capacity of both SIRs for V(V) approximately presents logarithmic growth with the increasing contacting time. The adsorption capacity of SIRs-CI reaches equilibrium at 10 h as 46.5 mg g^−1^, but that of SIRs-UI continuously increases after 10 h and finally arrives in equilibrium at about 14 h as 49.4 mg g^−1^, though the adsorption capacity of the former is always slightly higher than that of the latter in the first 10 h, which might also be attributed to the different existence form of the loaded extractant on two resins. From the working schematic of SIRs (figure 3), it is obviously observed that the adsorption process of metals onto SIRs involves the metal diffusion in aqueous phase, intraparticle diffusion in the resin and the reaction between the metal and extractant, of which the rate determining step is intraparticle diffusion in most cases [28]. Accordingly, the SIRs-UI with deeper extractant in the pores would need more time to arrive at the adsorption equilibrium with vanadium. Thus, the contacting time of 10 h is not enough to reach the adsorption equilibrium for SIRs-UI, which may lead to the slightly lower *Q*_M_ than that of SIRs-CI. It can be seen from figure 7*a*2 and *b*2 that vanadium distributes deeper in SIRs-UI than in SIRs-CI after adsorption of V(V), implying that the extractants reacting with V(V) can go deeper into the pores of the support under ultrasonic irradiation than in CI process, which also verifies that the extractant can go deeper into the pores of resins by ultrasound irradiation. It is obvious that the diffusion process of metal ions into the deeper pores will consume more time in comparison with that diffusing into the relative shallow places of the pores. Thus, the adsorption equilibrium time for the SIRs-UI may be longer than that for the SIRs-CI, which is consistent with the experimental results shown in figure 6, in terms of the fact that the equilibrium adsorption capacity for V(V) of SIRs-UI is higher than that of SIRs-CI, which is consistent to the fact that more N235 loaded on SIRs-UI can effectively react with V(V).

### 3.4 Adsorption of V(V) on the solvent-impregnated resins

**Figure 8.**
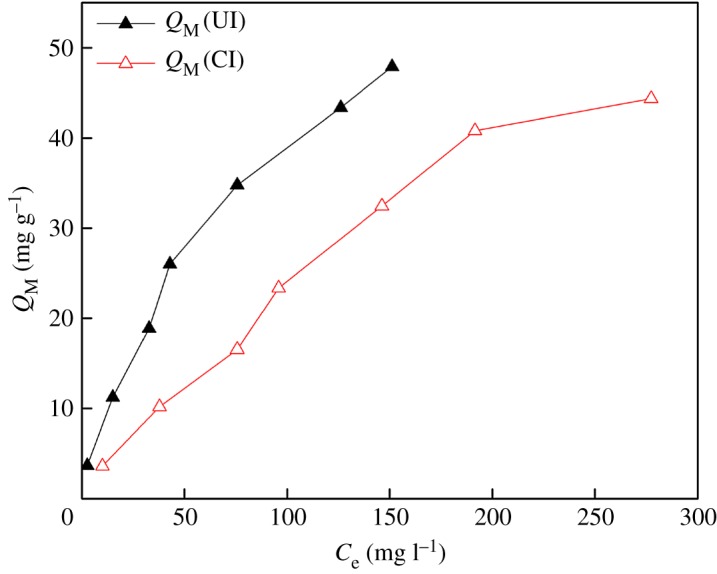
Effects of V(V) concentration on adsorption capacity of SIRs.

Figure 8 presents the effects of V(V) concentration on the adsorption capacity of SIRs-CI (contacting time 10 h) and SIRs-UI (contacting time 14 h) at the initial V(V) concentration of 100, 300, 500, 700, 950, 1200, 1350 mg l^−1^, respectively, by plotting *C*_e_ versus *Q*_M_.

As depicted in figure 8, the adsorption capacity of SIRs-UI and SIRs-CI for V(V) both increase with the increase of V(V) concentration. The equilibrium adsorption capacity of SIRs-UI for V(V) is always higher than that of SIRs-CI at the same initial V(V) concentration, which is consistent with the results concerning the adsorption capacity of SIRs for V(V) mentioned in section Impregnation time. The more homogeneously distributed extractant in the pores of SIRs-UI leads to higher adsorption capacity for V(V) onto such prepared SIRs.

#### 3.4.1. Adsorption isotherm

Generally, Langmuir and Freundlich isotherm models are used to depict the adsorption of metals onto SIRs. As is well known, the uptake of metal ions occurring on a homogeneous surface by monolayer adsorption can fit well the Langmuir isotherm model [28,29] which is listed as follows:

3.1 $$\frac{C_{e}}{Q_{e}}=\frac{1}{Q_{m}K_{L}}+\frac{C_{e}}{Q_{m}},$$

where *Q*_e_ is the amount of adsorbed metal ions per unit mass of adsorbent at equilibrium (mg g^−1^), *C*_e_ is the concentration of metal ions at equilibrium in raffinate (mg l^−1^) and *Q*_m_ is the adsorbent capacity (possible maximum amount of metal ions adsorbed per unit mass of adsorbent, mg g^−1^).

Freundlich model is based on the assumption that the adsorption of metals ions takes place on a heterogeneous surface by monolayer adsorption [14,29]. The linear form of this model is expressed as

3.2 $$\text{log}\;Q_{e}=\frac{1}{n}\text{log}\;C_{e}+\text{log}\;K_{F},$$

where *K*_F_ and *n* are the Freundlich constants for adsorption capacity and adsorption intensity, respectively.

**Figure 9.**
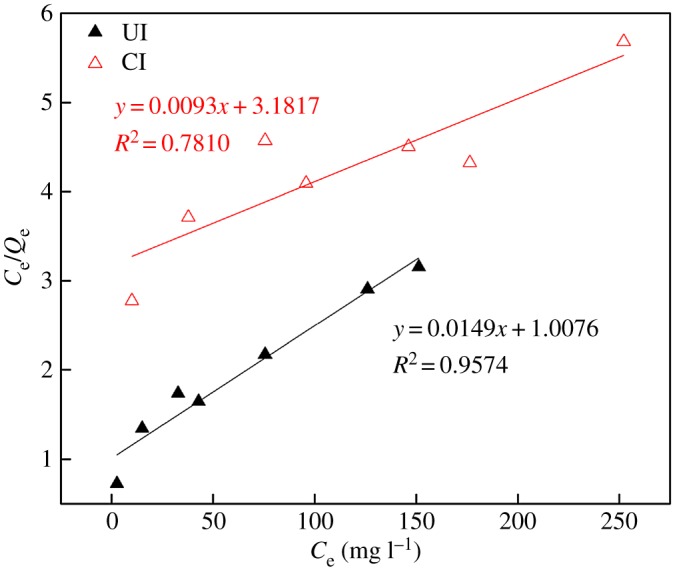
Langmuir adsorption isotherm of V(V).

**Figure 10.**
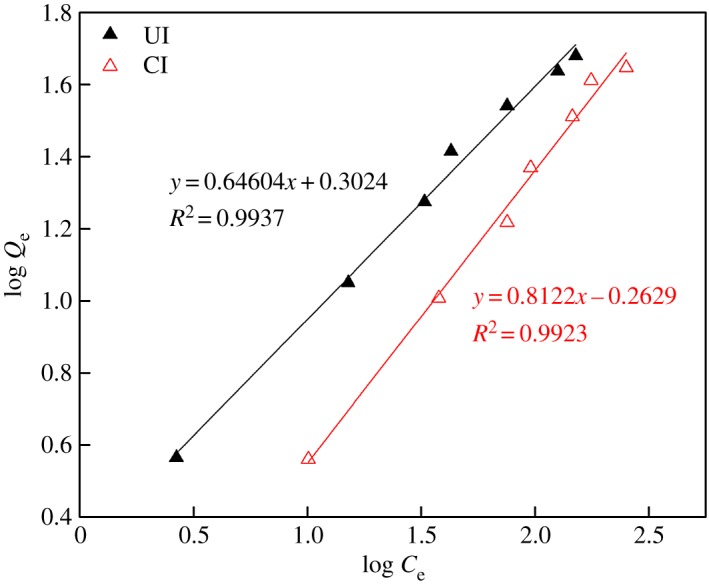
Freundlich adsorption isotherm of V(V).

The fitting of the adsorption of V(V) onto the SIRs according to Langmuir and Freundlich isotherm models are plotted in figures 9 and 10, respectively, and the corresponding fitting parameters are summarized in table 3.

**Table 3.** Parameters of the Langmuir and Freundlich isotherm models for V(V) adsorption.

	Langmuir			Freundlich		
impregnation methods	*Q*_m_	*K*_L_	*R*^2^	*n*	*K*_F_	*R*^2^
CI	107.5	0.0029	0.7810	1.2673	0.5856	0.9916
UI	67.11	0.0148	0.9574	1.5479	2.0063	0.9937

It is apparent that Freundlich isotherm model fits better to the adsorption of V(V) onto both SIRs than Langmuir isotherm model. The goodness of fit (*R*^2^) with Freundlich isotherm for SIRs-CI and SIRs-UI are 0.9916 and 0.9937, respectively, indicating that the Freundlich isotherm suits the system may be owing to the adsorption of V(V) on the SIRs not only in loaded N235 but also on the support resins [22]. It is obvious that the fitting parameters (*n* and *K*_F_) of Freundlich model for SIRs-UI are both larger than those for SIRs-CI, indicating V(V) is more easily absorbed on the SIRs-UI and the adsorption capacity of V(V) is higher than that of SIRs-CI, which is consistent with the experiment data.

### 3.5 Stability of the solvent-impregnated resins

The SIRs-UI and SIRs-CI were regenerated after adsorption so as to investigate the stability of those SIRs during cyclic use.

**Figure 11.**
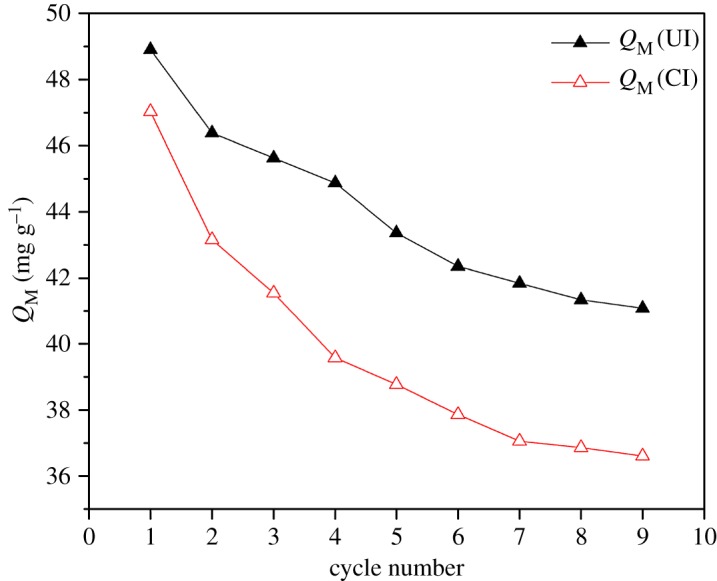
Adsorption capacity of SIRs for V(V) during cyclic use.

As can be seen from figure 11, the adsorption capacity of two SIRs for V(V) both decline with the adsorption–desorption cycles; however, the adsorption capacity of the SIRs-CI declines more obviously than that of SIRs-UI. The adsorption capacity of the SIRs-CI for V(V) is 77.83% of the initial value after the ninth cycle compared with 84.02% for the SIRs-UI, indicating that N235 loaded on the former is more likely to run off than that on the latter. This also verifies the existence of different forms of the extractant in the pores of two SIRs as discussed above. The extractant may be distributed more homogeneously [30] and deeper in the pores of SIRs-UI than in those of SIRs-CI, which endows SIRs-UI with stronger stability than SIRs-CI during the process of adsorption–desorption cycles.

## 4. Conclusion

Ultrasonic irradiation can enhance the impregnation of extractant onto the macroporous resins, but it makes the extractant enter deeper in the pores, resulting in the decline of utilization rate of the extractant with the increase of ultrasound power and the longer equilibrium time for adsorbing V(V). Moreover, ultrasonic irradiation also can promote the mass transfer and spread of extractant in the pores of resins and then largely reduce the impregnation equilibrium time compared with CI. Under appropriate ultrasound intensity, extractant is distributed more homogeneously in contrast to that in SIRs-CI, where the extractant prefers to accumulate in the pores to form multilayer films. Thus, SIRs-UI presents higher adsorption capacity for V(V) and utilization rate of extractant than SIRs-CI although the former impregnation rate is slightly lower than that of the latter, which may be due to the part desorption of extractant on the SIRs-UI caused by ultrasound effects. Ultrasonic irradiation may cause the different adsorption–desorption equilibrium for petroleum ether and N235 and thus leads to the low adsorption capacity of SIRs-UI for V(V) though SIRs-UI presents similar impregnation rate to SIRs-CI at low N235 content. The adsorption processes of V(V) onto two types of SIRs, SIRs-UI and SIRs-CI, fit Freundlich isotherm model, indicating that the adsorption of V(V) onto both SIRs is heterogeneous adsorption. The cyclic adsorption–desorption experiments show that the adsorption capacity of SIRs-CI for V(V) declines more obviously than that of SIRs-UI after nine cycles, indicating that SIRs-UI has stronger stability than SIRs-CI.

In sum, ultrasonic irradiation can impose many positive effects on the SIRs, such as enhancing mass transfer of the extractant, improving adsorption capacity for metals and strengthening the stability of the SIRs by changing the existence form of the extractant in resins pores. Ultrasound-assisted impregnation may be a potential and promising technique for the preparation of SIRs.

## Data accessibility

This article does not contain any additional data.
